# Safety and Effectiveness of Oral Anticoagulants in Atrial Fibrillation: Real-World Insights Using Natural Language Processing and Machine Learning

**DOI:** 10.3390/jcm13206226

**Published:** 2024-10-18

**Authors:** Juan Cosín-Sales, Manuel Anguita Sánchez, Carmen Suárez, Carlos Arias-Cabrales, Luisa Martínez-Sanchez, Savana Research Group, Daniel Arumi, Susana Fernández de Cabo

**Affiliations:** 1 Cardiology Department, Arnau de Vilanova Hospital, 46015 Valencia, Spain; 2 Faculty of Medicine, CEU-Cardenal Herrera University, Alfara del Patriaca, 46115 Valencia, Spain; 3 Clinical Management Unit of Cardiology, Reina Sofía University Hospital, 14004 Córdoba, Spain; manuelanguita@secardiologia.es; 4 Maimonides Institute for Biomedical Research of Córdoba (IMIBIC), University of Córdoba, 14004 Córdoba, Spain; 5 Biomedical Research Networking Center in Cardiovascular Diseases (CIBERCV), 28029 Madrid, Spain; 6 La Princesa University Hospital, 28006 Madrid, Spain; 7 La Princesa University Hospital Health Research Institute, 28006 Madrid, Spain; 8 Faculty of Medicine, Universidad Autónoma of Madrid, 28049 Madrid, Spain; 9 Savana Research S.L., 28010 Madrid, Spain; 10 Medical Department, Pfizer España, 28050 Madrid, Spain

**Keywords:** atrial fibrillation, oral anticoagulation, direct oral anticoagulants, vitamin K antagonist, natural language processing and machine learning

## Abstract

**Background/Objectives:** We assessed the effectiveness and safety of vitamin K antagonists (VKAs) versus direct oral anticoagulants (DOACs) in patients with atrial fibrillation (AF) using artificial intelligence techniques. **Methods:** This is a retrospective study in 15 Spanish hospitals (2014–2020), including adult AF patients with no history of anticoagulation, thrombosis events, rheumatic mitral valvular heart disease, mitral valve stenosis, or pregnancy. We employed EHRead^®^ technology based on natural language processing (NLP) and machine learning (ML), along with SNOMED-CT terminology, to extract clinical data from electronic health records (EHRs). Using propensity score matching (PSM), the effectiveness, safety, and hospital mortality of VKAs versus DOACs were analyzed through Kaplan–Meier curves and Cox regression. **Results:** Out of 138,773,332 EHRs from 4.6 million individuals evaluated, 44,292 patients were included, 79.6% on VKAs and 20.4% on DOACs. Most patients were elderly [VKA 78 (70, 84) and DOAC 75 (66, 83) years], with numerous comorbidities (75.5% and 70.2% hypertension, 47.2% and 39.9% diabetes, and 40.3% and 34.8% heart failure, respectively). Additionally, 60.4% of VKA and 48.7% of DOAC users had a CHA2DS2-VASc Score ≥4. After PSM, 8929 patients per subgroup were selected. DOAC users showed a lower risk of thrombotic events [HR 0.81 (95% CI 0.70–0.94)], minor bleeding [HR 0.89 (95% CI 0.83–0.96)], and mortality [HR 0.80 (95% CI 0.69–0.92)]. **Conclusions:** Applying NLP and ML, we generated valuable real-world evidence on anticoagulated AF patients in Spain. Even in complex populations, DOACs have demonstrated a better safety and effectiveness profile than VKAs.

## 1. Introduction

Atrial fibrillation (AF) is the most common cardiac arrhythmia globally, significantly increasing the risk of stroke or transient ischemic attack (TIA), emboli, and all-cause mortality [1]. Specifically, AF is accountable for an estimated 15% of strokes worldwide [2] and can be newly identified in about one-fourth of individuals post-stroke or post-TIA [3]. Consequently, the prevention of stroke and systemic embolism is fundamental in managing AF [4]. Currently, oral anticoagulants (OACs), including both vitamin K antagonists (VKAs) and direct-acting oral anticoagulants (DOACs), are the cornerstone of this prevention strategy.

DOACs have demonstrated a higher reduction in the relative risk of stroke/systemic embolism (SE), all-cause mortality, and intracranial hemorrhage compared to VKAs and are recommended as first-line medications in clinical guidelines [4,5]. However, despite the clinical guideline’s recommendations, there is a preference for prescribing VKAs over DOACs in Spanish real-world scenarios. This phenomenon arises from restrictions on DOAC prescriptions imposed by healthcare regulatory bodies, coupled with a lower rate of prescription in high-risk sub-populations characterized by multiple chronic conditions, polypharmacy, and high risk of bleeding and thrombosis [6]. First, a significant barrier in Spain is the requirement for a pre-approval process, which is an additional authorization process conducted by the health inspection services before DOACs can be prescribed, even with a medical prescription. Additionally, the higher financial cost of DOACs compared to VKAs further limits their widespread use. Second, this trend may be attributed to the underrepresentation of these sub-populations in clinical trials [7,8]. In this regard, real-world populations are more representative of clinical practice. Although the higher effectiveness and safety of DOACs compared to VKAs have also been confirmed in real-world studies [9,10,11,12], many of them rely on claims-based databases or International Classification of Diseases (ICD) codes, which might not accurately capture the clinical reality of OAC treatment for AF. Evidence from real-life observational studies on comparative effectiveness and safety outcomes in the Spanish population is limited, with only a single study focusing on apixaban [13]. Analyzing large volumes of real-world data (RWD) using natural language processing (NLP) and machine learning (ML) techniques to extract and analyze free text from electronic health records (EHRs) offers significant potential for a more accurate assessment of clinical data in routine clinical practice [14]. Using this novel technology to generate real-world evidence (RWE) regarding the effectiveness and safety of VKAs and DOACs could be useful for the decision-making process, especially in high-risk patients with AF.

The current research was conducted as part of the extensive AFIRMA Study (AF In Real practice on Management of oral Anticoagulation), which aimed to delineate the demographic and clinical characteristics of AF patients receiving VKAs and DOACs in Spain. Here, our objective was to compare effectiveness and safety outcomes and identify any significant differences in efficacy on thrombotic prevention, bleeding risk, and mortality between these two classes of OACs.

## 2. Materials and Methods

Study design and population. This multicenter and retrospective study utilized RWD from AF patients’ EHRs across 15 Spanish hospitals from the National Healthcare Network, including 7 country regions (Supplementary Table S1). Among all patients attended to in the participating hospitals during the study period, from January 2014 to December 2020, we selected those with at least one unambiguous mention of AF. Exclusion criteria were applied to define the final study population and encompassed those patients younger than 18 years with rheumatic mitral valvular disease (a leading cause of mitral stenosis), diagnosed with venous thromboembolism, pregnancy, undertaking previous anticoagulant therapy, or who had experienced a stroke, TIA, or SE within one month before inclusion. Additionally, we excluded patients whose inclusion date was later than 29 February 2020 to reduce the impact of COVID-19 on thrombotic event analysis and those patients with no follow-up data. Patients were stratified by OAC type into VKAs and DOACs, and a cross-sectional analysis was conducted at the inclusion date. Incident events of outcomes and hospital mortality were analyzed during the follow-up period, ranging from the inclusion date to the latest EHR within the study period.

Data source and extraction. Clinical data from EHRs were gathered from all departments of each site, including outpatient clinics, hospitalization, discharge summaries, and other reports after cleaning and pre-processing procedures. Both unstructured and structured data (when available) were extracted and analyzed using the EHRead^®^ technology (Savana, Madrid, Spain) described elsewhere [15], which is based on NLP and ML techniques to convert free text from EHRs into a study database [16,17]. The used terminology encompassed codes, synonyms, and definitions from clinical documentation based on SNOMED CT [17] and was internally validated. Moreover, we employed an NLP pipeline model based on ML that served not only to extract clinical entities but also to capture their pertinent attributes to detect their context. Further details on EHRead^®^ technology are provided in the Supplemental Methods.

EHRead^®^ performance was externally validated as previously described [15,18]. Briefly, expert physicians from each hospital annotated a random selection of EHRs containing study variables to set a comparison standard. These external annotations were compared with the variables detected by EHRead^®^ in the same EHR corpus. Further details regarding EHRead^®^ performance evaluation and specific metrics obtained are shown in the Supplemental Methods and in Table 1.

Study Variables. The study variables were extracted and analyzed as part of a curation process that guaranteed the quality and integrity of the data. Sociodemographic data, including age and sex, along with clinical variables such as comorbidities and medications, were collected. This information, including data from EHRs prior to the study start date, was used to reconstruct patient histories based on available records. Of note, concepts and their definitions were retrieved to the extent they were correctly registered in the included EHRs. Stroke/SE/TIA events were only considered if they were documented in at least two different sources. Stroke events included both ischemic and hemorrhagic types, whereas SE events excluded amniotic and gaseous embolisms. To identify major bleeding events, we adopted a comprehensive definition that integrates criteria from the International Society on Thrombosis and Haemostasis [19], implementing those pertaining to clinically relevant bleeding [20]. Accordingly, specific definitions for major bleeding were established and are detailed in the Supplemental Methods. Bleeding events not meeting the major bleeding criteria were classified as minor, including cases of gastrointestinal bleeding without the need for transfusion. Death events were recorded solely from the free text available in the EHRs. For variables analyzed around discrete time points, the closest value to the time point (within reference time windows) was taken. Reference time windows accounted for the variability in healthcare management between patients, specialists, and hospitals, maximizing data retrieval from EHRs. Time window ranges for each variable or group of variables are detailed in table footnotes.

Statistical Analyses. Categorical and binary variables are summarized by frequencies, numerical variables by median, quartile 1, quartile 3 (Q1, Q3), and available value counts. The number of patients with evaluable data is shown in the analysis for discrete and continuous variables. Binary variables like comorbidities or symptoms were considered absent if not recorded. Multi-level categorical variables, lifestyle factors, and numeric variables were not imputed. Propensity score matching (PSM) was used to pair patients (VKAs/DOACs) on a wide range of confounders, as identified in a previous study [12]. Those included year of inclusion, bleeding history, hypertension, diabetes, renal disease, sex, transient ischemic accident, anemia, stroke history, thrombocytopenia, age, peripheral vascular disease, myocardial infarction, coronary disease, cirrhosis, alcohol use, heart failure, dyspepsia, multi-morbidity index, and medication use (antiplatelets, insulin, angiotensin-converting enzyme inhibitors [ACEs], angiotensin receptor blockers [ARBs], oral antidiabetic drugs, lipid-lowering drugs, beta-blocking agents, and non-steroidal anti-inflammatory drugs). The nearest-neighbor matching method without replacement with a caliper of 0.2 standard deviation [21] was used. Covariate balance was verified using Love plots based on observed standardized mean differences with a threshold of 10%. Kaplan–Meier curves illustrated treatment group outcomes, with univariate Cox regression comparing survival across anticoagulant treatments, using VKAs as a reference. Additionally, absolute risk differences in outcome development at five years between the DOAC and VKA groups were calculated based on Kaplan–Meier estimates. Goodness of fit for the univariate Cox model was assessed using the Schoenfeld residuals test to identify any significant trends suggesting a violation of the proportional hazards assumption. Moreover, two sensitivity analyses were conducted. First, a sensitivity analysis was performed by restricting the follow-up period to 3 years to help balance the follow-up period between the cohorts. Second, multivariate Cox proportional hazard models were conducted on all patients meeting the eligibility criteria (without PSM but with all covariates previously used for propensity score estimation alternatively adjusted for in the Cox models). Significance was determined by a *p*-value <0.05 and a 95% CI. Analysis was performed using “R” software (version 4.0.2). Details on how missing values were handled are provided in the Supplementary Materials.

## 3. Results

### 3.1. Study Population

**Figure 1:**
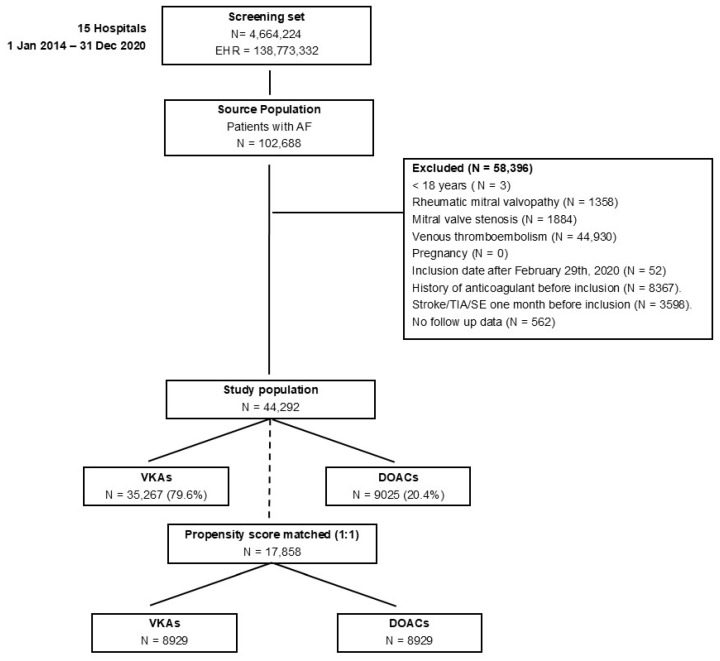
Flow chart with an overview of the patient selection methodology. The initial screening set for our study comprised over 4.6 million patients at participating hospitals, processing nearly 139 million EHRs. The application of exclusion filters (age <18 years, rheumatic mitral valvopathy, mitral valve stenosis, venous thromboembolism, pregnancy, previous anticoagulant therapy, recent stroke, TIA or SE, no follow-up data, and the cutoff date to mitigate COVID-19 impacts) defined a study population of 44,292 patients with atrial fibrillation (AF), with 35,267 (79.6%) on VKAs and 9025 (20.4%) on DOACs. Patients on VKAs and DOACs were matched using propensity score matching (PSM) to ensure balanced groups for comparative analysis.

A total of 138,773,332 EHRs from 4,664,224 patients attended to in the participating hospitals during the study period were processed. Within these, 102,688 patients had AF, and 44,292 were finally included in the study population, with 35,267 (79.6%) receiving VKAs and 9025 (20.4%) receiving DOACs (Figure 1). The median (Q1, Q3) age of patients was 78 (69, 84) years in the overall population (78 (70, 84) in the VKA group and 75 (66, 83) in the DOAC group). Males were 53% overall (52% in the VKA group and 56% in the DOAC group (Table 2)). Cardiovascular comorbidities were frequent among the included patients; hypertension was reported in 75.5% and 70.2% of VKA and DOAC patients, respectively; diabetes mellitus in 47.2% and 39.9%; and heart failure in 40.3% and 34.8%. Other relevant comorbidities, such as renal disease or history of bleeding, were reported in 18.7% and 25.1% of VKA patients and 14.6% and 20.2% in the DOAC group, respectively. Additionally, 18.2% of VKA and 13.3% of DOAC patients had a multi-comorbidity index ≥3, which means they presented at least three concurrent pathologies. Regarding concomitant treatments, beta-blocking agents, ACEs or ARBs, and diuretics were the most frequently reported both in VKA and DOAC patients. Additionally, a high CHA2DS2-VASc Score (4+) was reported in 60.4% of patients on VKAs and 48.7% in the DOAC group (Table 2).

### 3.2. Incidence Rates of Effectiveness, Safety, and Hospital Mortality

The median (Q1, Q3) follow-up time was 2.7 (1.3, 4.0) years. During this period, we registered 2548 (5.7%) cases of stroke/SE/TIA, with an overall incidence rate of 2.2 per 100 person-years. These typically occurred at a median (Q1, Q3) of 0.8 (0.1, 2.0) years after the first mention of AF (inclusion date). Major bleeding was documented in 4178 (9.4%) participants, with an incidence rate of 3.7 per 100 person-years, and minor bleeding events in 10,412 (23.5%) patients. There were 2988 (6.7%) deaths reported, corresponding to an incidence rate of 2.5 per 100 persons-years. The distribution and incidence rates of these events among VKA and DOAC users are presented in Table 3.

### 3.3. Comparison Rates of Effectiveness, Safety, and Hospital Mortality

**Figure 2:**
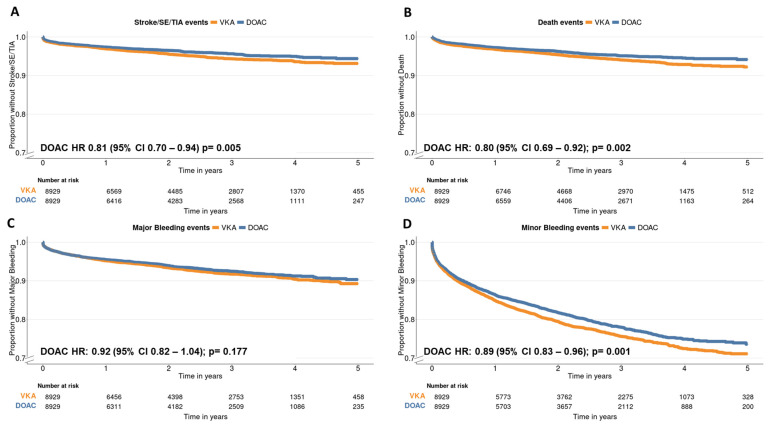
Kaplan–Meier curves for PSM patients comparing DOACs and VKAs showing treatment outcomes for stroke/SE/TIA (**A**), death (**B**), major (**C**), and minor bleeding (**D**). Cox regression analysis was employed to compare survival across treatments, with VKAs serving as the reference. Significance was determined based on a *p*-value < 0.05 and a 95% confidence interval. The proportion y-axis has been adjusted to 0.7 for better visualization of results. See Supplemental results (Supplementary Figure S3) for non-adjusted curves.

After PSM, up to 8929 patients from each group, VKAs and DOACs, were analyzed (Figure 1). The Love plot indicated a correct covariate balance, with a standardized mean difference lower than 10% for all confounders (Supplementary Figure S1). Patients on DOACs experienced fewer stroke/SE/TIA events than those on VKAs, with a significantly lower risk for its development (HR: 0.81, 95% CI 0.70–0.94; *p* = 0.005), as illustrated in Figure 2A. Patients on DOACs also exhibited a lower proportion and risk of death compared to those in the VKA group (HR: 0.80, 95% CI 0.69–0.92; *p* = 0.002), as depicted in Figure 2B. There were no differences between DOAC and VKA patients in major bleeding events (Figure 2C). However, the DOAC group presented a lower risk of minor bleeding events (HR: 0.89, 95% CI 0.83–0.96; *p* = 0.001, Figure 2D). The 5-year risk difference (comparing DOAC treatment vs VKA treatment) for stroke/SE/TIA was −1.31 per 100 persons (95% CI: −1.51,−1.11 per 100 persons); for death, it was −1.67 per 100 persons (95% CI: −3.15, 1.34 per 100 persons); for major bleeding, −1.10 per 100 persons (95% CI: −3.15, 1.34 per 100 persons); and for minor bleeding, −3.59 per 100 persons (95% CI: −3.96, −3.22 per 100 persons).

The results regarding the goodness-of-fit tests are shown in Supplementary Figure S2. After plotting the Schoenfeld residuals, we did not observe any significant trends, which suggests that the proportional hazards assumption was not violated. The Schoenfeld individual test yielded a *p*-value greater than 0.005 for all the outcomes, further supporting the validity of the proportional hazards assumption for treatment effect. These results indicate that the effect estimates from our model are reliable and consistent over time. In the two sensitivity analyses, the results were generally consistent with the main analysis (Supplementary Table S2).

## 4. Discussion

The AFIRMA study marks the first initiative to assess the effectiveness and safety outcomes of OAC treatment in a real-world population of AF patients, employing NLP and ML to extract and analyze RWD. Leveraging this technology enabled us to construct a comprehensive and detailed patient cohort, elucidating the clinical profile of these patients in our context. Moreover, it provided us with estimates of the incidence rates for clinically significant events. Ultimately, this approach allowed us to conduct a paired comparison between the DOAC and VKA treatments, considering an extensive list of confounders. Our findings indicate that DOACs are associated with higher effectiveness and lower hospital mortality compared to VKAs, with no significant differences observed in major bleeding events.

Our results highlight a high-risk profile among patients with AF in our setting, characterized by an elderly population with a high burden of comorbidities and a significant risk of thrombosis, as reflected by elevated values in the CHA2DS2-VASc Score. These findings are particularly relevant given that such patient profiles are underrepresented in clinical trials. For instance, a meta-analysis utilizing data from four phase 3 clinical trials that compared various DOACs with warfarin [5] disclosed that only 30–40% of participants were aged 75 years or older, and approximately half possessed a CHA2DS2-VASc Score of 3 or higher. Conversely, our study identified a median age of 78 years, with nearly 80% of participants presenting CHA2DS2-VASc Score values of the same magnitude.

Our study reveals that patients treated with DOACs exhibited lower incidence rates of stroke/SE/TIA, safety outcomes (including both major and minor bleeding events), and hospital mortality compared to those on VKAs. Notably, this difference was confirmed after PSM—excluding major bleeding events, where differences were observed but not significant—enabling us to assess the independent effect of treatment on these outcomes. Specifically, over a period of five years, for every 100 patients treated with DOACs instead of VKAs, we found that DOACs reduce the incidence of stroke/SE/TIA by approximately 1.31 cases, mortality by 1.67 cases, and minor bleeding by 3.59 cases, without increasing the risk of bleeding. The favorable risk–benefit profile of DOACs, with significant reductions in stroke, intracranial hemorrhage, and mortality, has been reported in both pivotal clinical trials and RWD studies [5,11,22]. However, the outcomes regarding bleeding risks (major, gastrointestinal, and any bleeding) vary depending on the specific DOAC evaluated. In this regard, dabigatran and apixaban have been associated with a lower risk of major bleeding compared to VKAs, while VKAs showed a lower risk than rivaroxaban for this particular outcome. Regarding gastrointestinal bleeding, VKAs exhibited a lower risk when compared to both rivaroxaban and dabigatran; however, apixaban demonstrated superiority over VKAs in this sense. The findings on any bleeding events also varied; some DOACs showed reduced risk of bleeding compared to VKAs, while the risk with other DOACs was found to be similar [5,11,22]. Unfortunately, we did not perform comparative analyses of specific DOACs and different types of bleeding, preventing us from concluding whether the non-superiority of DOACs against VKAs in terms of major bleeding could be attributed to specific drugs or types of bleeding episodes.

This multicenter study is distinguished by its use of advanced technology to compile an extensive dataset of Spanish AF patients receiving OAC treatment, making it the most comprehensive to date. Previous studies have used RWD to characterize or evaluate patients with AF treated with VKAs or DOACs in different European countries [23,24,25]. Importantly, our innovative approach based on NLP and ML techniques enabled the extraction of critical data from unstructured free-text narratives, significantly enhancing the data’s richness beyond that of previous real-life studies. Additionally, our methodology offers significant advantages over clinical trials or studies based on claims data, which often suffer from selection biases due to strict inclusion and exclusion criteria, limiting the generalizability of their findings. Our approach allows for a more inclusive and representative sample of the patient population. However, this study has some limitations. Owing to its retrospective nature and reliance on RWD, our study faced challenges such as missing important variables due to underreporting in electronic health records (EHRs), including international normalized ratio (INR) values. A notable limitation is the identification of mortality cases solely through clinical records in the hospital setting. In this regard, access to official mortality databases was not possible due to data anonymization, potentially leading to an underestimation of this outcome. Furthermore, the absence of comparative analyses by specific DOAC types and subtypes of major bleeding represents a limitation to deepening our understanding of treatment impacts. Additionally, relying exclusively on hospital-sourced data may not capture the full spectrum of AF patients on OACs in outpatient settings, potentially limiting the generalizability of our findings to a broader population. Finally, we were unable to perform a post hoc quality assurance on a random sample of our results due to regulatory constraints to ensure data confidentiality.

In summary, the AFIRMA study employed NLP and ML to generate a comprehensive database including RWD extracted from patients’ EHRs, offering profound RWE insights into OAC therapy for Spanish patients with AF. It highlights the superior effectiveness and comparable safety profile of direct DOACs compared to VKAs, even in a patient cohort characterized by a high burden of comorbidities and elevated risk of thrombosis, populations often underrepresented in clinical trials. Consequently, our study bridges a critical gap in the evidence base, providing invaluable guidance for the optimization of AF management in clinical practice and advocating for a more personalized, evidence-based approach to selecting treatments. Future research should explore outcomes based on specific DOAC types and various bleeding subtypes. Additionally, it is crucial to examine the potential policy implications of our findings, particularly regarding the under-prescription of DOACs in Spain. By addressing these issues, our study could help drive changes in clinical practice, ultimately improving patient outcomes and aligning treatment approaches with clinical guidelines.

## Figures and Tables

**Table 1:** Reading performance of EHRead^®^ Technology.

Variable	Precision	Recall	F1-Score
Hemoglobin	0.97	0.92	0.94
Atrial fibrillation	0.97	0.83	0.89
Transient ischemic attack	0.91	0.82	0.86
Intracranial hemorrhage	0.87	0.55	0.67
Bleeding	0.74	0.73	0.74
Transfusion	0.73	0.95	0.83
Treatments			
Dabigatran	0.99	0.94	0.96
Edoxaban	0.99	091	0.95
Rivaroxaban	0.99	0.93	0.96
Warfarin	0.99	0.91	0.95
Acenocumarol	0.98	0.90	0.93
Apixaban	0.95	0.96	0.95

**Table 2:** Patient demographic, clinical characteristics, and concomitant treatment at inclusion.

	Overall (n = 44,292)	VKAs (n = 35,267)	DOACs (n = 9025)
Age, years, median (Q1, Q3)	78 (69, 84)	78 (70, 84)	75 (66, 83)
Male sex, n (%)	23,304 (53)	18,221 (52)	5083 (56)
Alcohol abuse, n (%)	2142 (4.8)	1792 (5.1)	350 (3.8)
Comorbidities, n (%)			
Hypertension *	32,954 (74.4)	26,615 (75.5)	6339 (70.2)
Diabetes mellitus *	20,262 (45.7)	16,660 (47.2)	3602 (39.9)
Heart failure *	17,348 (39.2)	14,208 (40.3)	3140 (34.8)
Anemia	13,849 (31.3)	11,841 (33.6)	2008 (22.2)
History of bleeding	10,683 (24.1)	8862 (25.1)	1821 (20.2)
Renal disease *	7923 (17.9)	6609 (18.7)	1314 (14.6)
Coronary disease *	4048 (9.1)	3273 (9.3)	775 (8.6)
Ischemic stroke *	1919 (4.3)	1507 (4.3)	412 (4.6)
Myocardial infarction *	1297 (2.9)	1057 (3.0)	240 (2.7)
TIA *	1063 (2.4)	864 (2.4)	199 (2.2)
Thrombocytopenia	978 (2.2)	828 (2.3)	150 (1.7)
Dyspepsia	654 (1.5)	522 (1.5)	132 (1.5)
Cirrhosis *	528 (1.2)	446 (1.3)	82 (0.9)
Peripheral vascular disease *	123 (0.3)	102 (0.3)	21 (0.2)
Multi-comorbidity index ≥3, n (%) †	7609 (17.2)	6405 (18.2)	1204 (13.3)
Concomitant treatment, n (%)			
Beta-blocking agents	22,798 (51.5)	17,847 (50.6)	4951 (54.9)
ACEs or ARBs	22,080 (49.9)	17,955 (50.9)	4125 (45.7)
Diuretics	20,459 (46.2)	17,029 (48.3)	3430 (38.0)
Antiplatelets	9332 (21.1)	7547 (21.4)	1785 (19.8)
OADs	6121 (13.8)	4963 (14.1)	1158 (12.8)
NSAIDs	4706 (10.6)	3779 (10.7)	927 (10.3)
Insulin and analogs	3449 (7.8)	2930 (8.3)	519 (5.8)
Lipid-lowering agents	2249 (5.1)	1781 (5.1)	468 (5.2)
CHA2DS2-VASc Score, n (%)			
4+	25,699 (58.0)	21,301 (60.4)	4398 (48.7)
3	9103 (20.5)	7175 (20.3)	1928 (21.3)
2	5542 (12.5)	4171 (11.8)	1371 (15.2)
0–1	3669 (8.2)	2419 (6.8)	1250 (13.8)

VKAs: vitamin K antagonists; DOACs: direct oral anticoagulants; ACEs: angiotensin-converting enzyme inhibitors; ARBs: angiotensin receptor blockers; NSAIDs: non-steroidal anti-inflammatory drugs; OADs: oral antidiabetic drugs; TIA: transient ischemic attack; Q1: first quartile; Q3: third quartile; * variables used for the multi-comorbidity index calculation; † the multi-comorbidity index is a binary variable in which each comorbidity is included as +1. The index is considered negative when its value is <3, and positive when ≥3. Time window considered: (first report, inclusion date).

**Table 3:** Incidence Rates of stroke/SE/TIA, major bleeding, minor bleeding, and death events in patients treated with OACs (overall and by OAC group).

	Overall (n = 44,292)	VKAs (n = 35,267)	DOACs (n = 9025)
Follow-up, years, median (Q1, Q3)	2.7 (1.3, 4.0)	2.9 (1.5, 4.2)	2.0 (0.9, 3.3)
Effectiveness outcomes			
Stroke/SE/TIA, n (%)	2548 (5.7)	2234 (6.3)	314 (3.5)
Incidence Rate (n per 100 per-years)	2.2	2.3	1.6
Time to event, median (Q1, Q3)	0.7 (0.1, 1.8)	0.8 (0.1, 1.9)	0.4 (0.1, 1.3)
Safety outcomes			
Major Bleeding, n (%)	4178 (9.4)	3638 (10.3)	540 (6.0)
Incidence rate (n per 100 per-years)	3.7	3.9	2.9
Time to event, median (Q1, Q3)	0.9 (0.2, 2.1)	0.9 (0.2, 2.2)	0.6 (0.1, 1.8)
Minor Bleeding, n (%)	10,412 (23.5)	8838 (25.1)	1574 (17.4)
Incidence rate (n per 100 per-years)	10.6	10.9	9.4
Time to event, median (Q1, Q3)	0.6 (0.1, 1.7)	0.7 (0.1, 1.7)	0.5 (0.1, 1.5)
Mortality			
Deaths, n (%)	2988 (6.7)	2652 (7.5)	336 (3.7)
Incidence rate (n per 100 per-years)	2.5	2.6	1.7
Time to event, median (Q1, Q3)	0.8 (0.2, 1.9)	0.8 (0.2, 2.0)	0.5 (0.1, 1.4)

VKAs: vitamin K antagonists; DOACs: direct oral anticoagulants; Q1: first quartile; Q3: third quartile. Time-to-event results are reported in years. Time window considered: (inclusion date, latest EHR).

## Data Availability

The data underlying this article were provided to the authors by permission of the affiliated institutions. Data can be shared on reasonable request to the corresponding author after permission has been obtained from the institutions involved.

## Supplementary Materials

The following supporting information can be downloaded at https://www.mdpi.com/article/10.3390/jcm13206226/s1: Supplemental Methods; Table S1. Participating centers by region; Table S2. Sensitivity analysis; Figure S1. Love plot: evaluation of covariate balance after PSM; Figure S2. Cox proportional hazards regression for PSM patients comparing DOACs and VKAs; Figure S3. Non-adjusted Kaplan–Meier curves for PSM patients comparing DOACs and VKAs showing treatment outcomes for stroke/SE/TIA (A), death (B), major (C), and minor bleeding (D).
