# Supplementary material for: Safety and Effectiveness of Oral Anticoagulants in Atrial Fibrillation: Real-World Insights Using Natural Language Processing and Machine Learning
Source: J Clin Med. 2024 Oct 18;13(20):6226. doi: 10.3390/jcm13206226 (PMC11508721; doi:10.3390/jcm13206226)
Supplement: Supplementary file 1 [file jcm-13-06226-s001.zip › jcm-3209235-supplementary.pdf]

Real-World Insights: Safety and Efficacy of Oral Anticoagulants in Nonvalvular Atrial Fibrillation Leveraging Natural Language Processing and Machine Learning

SUPPLEMENTAL MATERIALS

SUPPLEMENTAL METHODS

This study was conducted under a rigorous study protocol and a statistical analysis plan to ensure that the data were “fit-for-purpose” and that our analysis approach was pre-specified and systematic. This framework guided the design of our data extraction strategy from each hospital, the engineering of the NLP processing, and the execution of the appropriate analyses to meet the study objectives.

Data source

We created specific documentation based on the study protocol, detailing the required data sources. This documentation specified the data form (structured, semi-structured, free-text, etc.) and the type of data (e.g., hospital departments, hospital areas such as emergency rooms or outpatient clinics) expected from the participating hospitals. Once the sites were included in the study, we generated an initial data quality report to confirm the availability and number of patient/records during the study period for each hospital, with a focus on key departments and report types. Only those sites that met all the criteria described were included in the research study. After the complete data integration process, we performed specific technical and clinical checks to detect non-informative reports or data inconsistencies (such as a significant percentage of pediatric patients in the study population) to be further removed or reviewed if needed. Following these checks, the data were cleaned and prepared for variable extraction.

Extraction of the Unstructured Information from Electronic Health Records

All study variables were extracted from patients Electronic Health Records (EHRs) using EHRead® technology which uses Natural language processing (NLP) and Machine learning (ML) techniques for extracting and translating free text into a study database. This process required that first conceptual definitions for all study variables were pre-specified and aligned with clinical entities found in the SNOMED Clinical Terms (a comprehensive, computationally processable collection of medical terms utilized in

clinical documentation) using the SNOMED CT browser. This step facilitated the conversion of unstructured data from various hospital departments into actionable variables for extraction. The clinical accuracy of the conceptual definitions and entity mapping was reviewed and approved by medical research experts specialized in NLP. For internal validation, medical research experts performed dedicated annotation projects and examined study variable detection summaries to identify false positives and false negatives, ensuring that the NLP modules designed to detect them performed as expected. In addition, if training data was required for specific ML models, dedicated annotation projects were created and annotated again by medical research experts trained in such tasks to provide the gold standard corpora. To judge the ML model performance, the performance metrics precision, recall and f1-score were calculated. Besides the study specific NLP modules, several general NLP modules were applied, which had undergone a rigorous quality check (e.g., negation detection, temporality). All clinical entities of the study were then extracted from pseudonymized EHRs using EHRead®, and variables were constructed by applying dedicated data wrangling operations to their mapped entities, leveraging specific NLP parameters generated by dedicated ML models (e.g., negation, temporality, attributes, etc.) and record-specific metadata (e.g., date, medical department, record type, etc.). The employed NLP pipeline models served not only to extract clinical entities but also to capture their pertinent attributes. The final output of EHRead® is a structured database that contains the full information about all detected clinical entities and their context, enabling downstream statistical analyses.

EHRead® performance

To ensure the quality of data extraction, the performance of EHRead® was externally evaluated. Specifically, this validation was carried out by external annotators following a peer-reviewed method [1]. Briefly, the external annotators created the 'standard' to which EHRead® technology's variable detections were compared. The aim was to measure

inter-annotator agreement (IAA) to ensure guideline consistency and parameter reliability, using these annotations as a benchmark to assess EHRead® against physician annotations.

Additionally, to determine the required minimum number of annotated EHRs, we employed the Sample Calculator for Evaluation (SLiCE®), a tool designed to calculate this based on the prevalence of key variables (in this case, AF and OAC) within the EHRs. SLiCE uses 95% confidence level interval widths of 10% (percentage points), and targets for precision and recall, ensuring that the estimated precision and recall are accurate within ±5% (percentage points) at a 95% confidence level.

The evaluation of the system was calculated in terms of the standard metrics of Precision, Recall, and their harmonic mean F1-Score.

- *Precision* = $\frac{tp}{tp + fp}$. This parameter indicates the accuracy of the system in retrieving key clinical concepts.
- *Recall* = $\frac{tp}{tp+fn}$. This parameter indicates the amount of information the system retrieves.
- *F1-Score* = $\frac{2xPrecisionxRecall}{Precision+Recall}$. This parameter gives us an overall performance indicator of information retrieval.

In all cases, *tp* is the number of true positives (i.e., records correctly retrieved), *fn* is the set of false negatives (i.e., records incorrectly not retrieved), and *fp* is the number of false positives (i.e., records incorrectly retrieved).

The results of EHRead® performance metrics for this study are shown in **Table S2**.

Major bleeding definition

Major bleeding was defined as any of the following:

- Fatal bleeding.
- Bleeding leading to death during hospital admission.

- A drop in hemoglobin >2 g/dL within 48 hours due to bleeding or surgery.
- Transfusion needs of ≥2 units of whole blood or red cells within 48 hours post-event.
- Bleedings in critical areas or organs, such as:
    - Primary hemorrhagic stroke.
    - Intracranial bleeding.
    - Intraocular bleeding.
    - Intraspinal bleeding.
    - Pericardial bleeding.
    - Retroperitoneal bleeding.
    - Gastrointestinal bleeding that required transfusion.

Management of missing values

For descriptive analyses, missing data were handled according to the nature of the data collection process assuming that physicians reflected clinically relevant information in EHRs. In this context, missing data imputation may occur for certain types of variables. For example, comorbidities and symptoms were treated as Boolean variables so that, the absence of a term referring to a specific comorbidity/symptom was treated in the same way as a negated comorbidity/symptom (i.e., the patient lacks that comorbidity/symptom). For multilevel categorical variables that may not be always reflected by physicians, such as lifestyle factors (e.g., smoking habits, marital status), their absence was not imputed and missing data was included in percentage calculations, unless otherwise specified. For numeric variables, no missing data imputation strategies were applied.

SUPPLEMENTAL RESULTS

Table S1. Participating centers by region

Center	Region
Hospital Universitario de La Princesa	Madrid
Hospital Universitario de Fuenlabrada	Madrid
Hospital Universitario Infanta Leonor	Madrid
Hospital Universitario Infanta Sofía	Madrid
Hospital Universitario Puerta de Hierro	Madrid
Hospital General Universitario de Castellón	Valencia
Hospital Clínico Universitario de Valencia	Valencia
Hospital Universitario y Politécnico La Fe	Valencia
Hospital de la Santa Creu i Sant Pau	Catalonia
Hospital Universitario Vall d´Hebron	Catalonia
Hospital de León	Castile and Leon
Hospital Río Hortega	Castile and Leon
Hospital Regional Universitario Carlos Haya	Andalusia
Hospital Universitario Son Espases	Balearic Islands
Clínica Universitaria de Navarra	Navarra

Table S2. Sensitivity Analysis.

	Stroke/SE/TIA	Death	Major Bleeding	Minor Bleeding
**Hazard ratios restricted by 3-years of follow up**				
HR ratio	0.79 (0.68-0.93, p=0.003)	0.82 (0.71-0.95, p=0.008)	0.92 (0.82-1.04, p=0.184)	0.89 (0.83-0.95, p=0.001)
**Multivariate Cox regression model**				
DOAC HR ratio	0.81 (0.72-0.91, p=0.001)	0.80 (0.71-0.89, p<0.001)	0.90 (0.82-0.98, p=0.022)	0.86 (0.81-0.91, p<0.001)

Figure S1. Love plot, evaluation of covariate balance after PSM

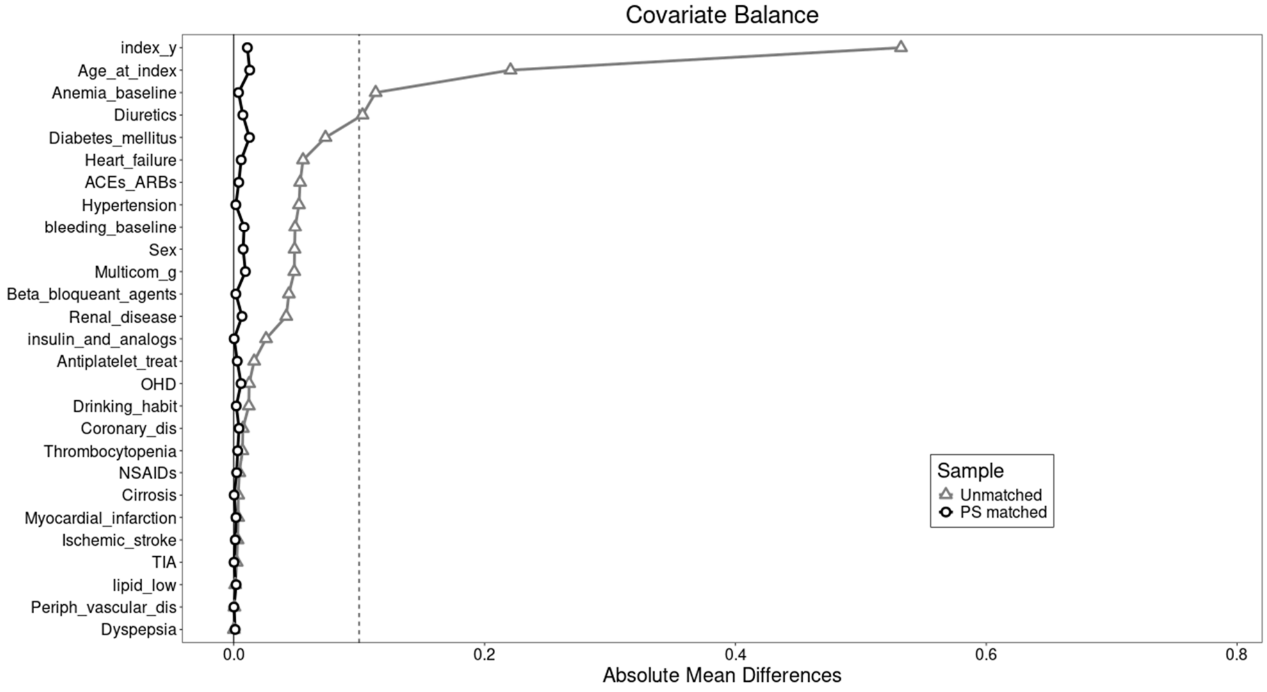

Figure S2. Cox proportional hazards regression for PSM patients comparing DOACs and VKAs

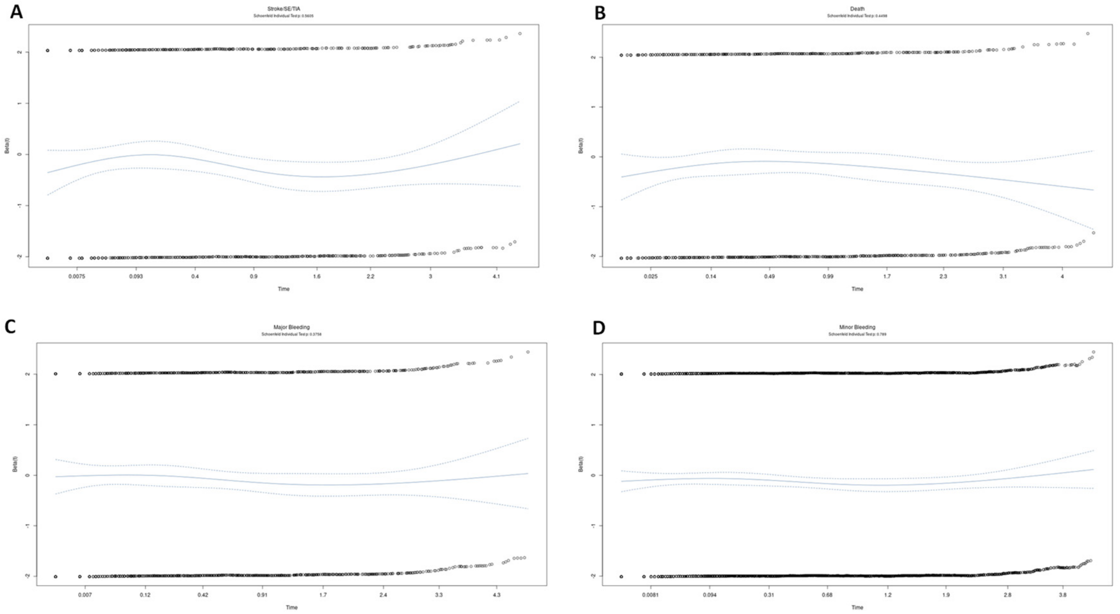

Figure S3. Non-adjusted Kaplan–Meier curves for PSM patients comparing DOACs and VKAs showing treatment outcomes for stroke/SE/TIA (A), death (B), major (C), and minor bleeding (D).

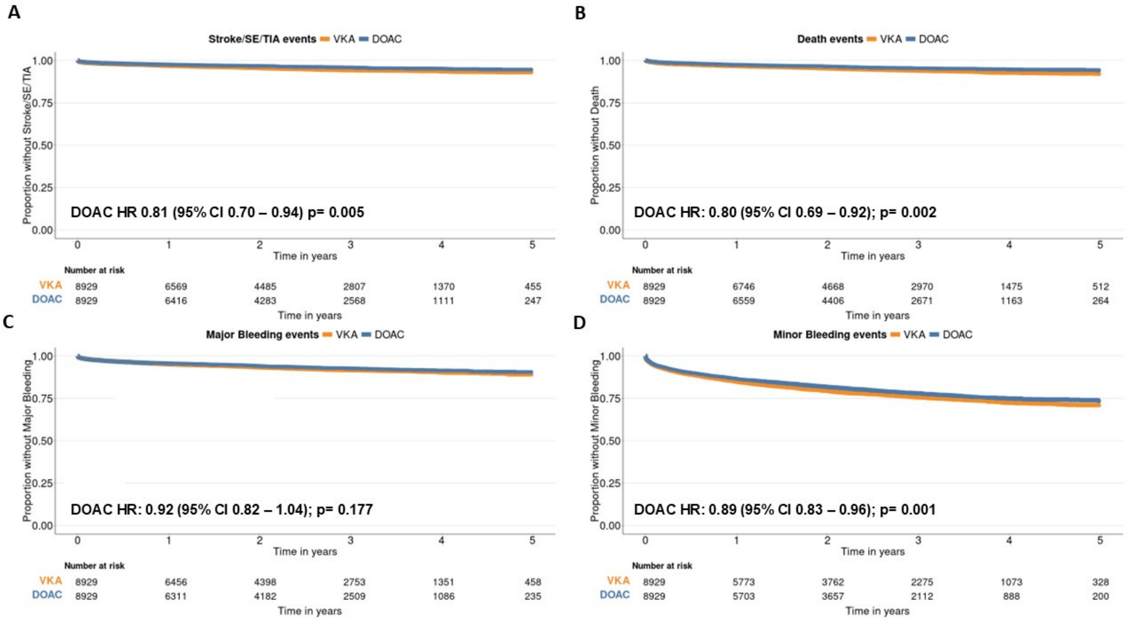

REFERENCES

1. Canales, L., et al., *Assessing the Performance of Clinical Natural Language Processing Systems: Development of an Evaluation Methodology.* JMIR Med Inform, 2021. **9**(7): p. e20492.
